# Hexokinase 2 is a molecular bridge linking telomerase and autophagy

**DOI:** 10.1371/journal.pone.0193182

**Published:** 2018-02-20

**Authors:** Jae-il Roh, Yujin Kim, Jahyun Oh, Yunmi Kim, Jeehyun Lee, Jaehoon Lee, Kyung-Hee Chun, Han-Woong Lee

**Affiliations:** 1 Department of Biochemistry, College of Life Science and Biotechnology and Yonsei Laboratory Animal Research Center, Yonsei University, Seoul, Republic of Korea; 2 Department of Biochemistry & Molecular Biology, Yonsei University College of Medicine, Seoul, Republic of Korea; Univerzitet u Beogradu, SERBIA

## Abstract

Autophagy is systematically regulated by upstream factors and nutrients. Recent studies reported that telomerase and hexokinase 2 [HK2) regulate autophagy through mTOR and that telomerase has the capacity to bind to the HK2 promoter. However, the molecular linkage among telomerase, HK2, and autophagy is not fully understood. Here, we show that HK2 connects telomerase to autophagy. HK2 inhibition in HepG2 cells suppressed TERT-induced autophagy activation and further enhancement by glucose deprivation. The HK2 downstream factor mTOR was responsible for the TERT-induced autophagy activation under glucose deprivation, implying that TERT promotes autophagy through an HK2-mTOR pathway. TERC played a role similar to that of TERT, and simultaneous expression of TERT and TERC synergistically enhanced HK2 expression and autophagy. At the gene level, TERT bound to the HK2 promoter at a specific region harboring the telomerase-responsive sequence ‘TTGGG.’ Mutagenesis of TERC and the TERT-responsive element in the HK2 promoter revealed that TERC is required for the binding of TERT to the HK2 promoter. We demonstrate the existence of a telomerase-HK2-mTOR-autophagy axis and suggest that inhibition of the interaction between telomerase and the HK2 promoter diminishes glucose starvation-induced autophagy.

## Introduction

Telomerase, a ribonucleoprotein complex of telomerase reverse transcriptase (TERT) and telomerase RNA component (TERC), is responsible for maintaining telomeres, which contain tandem repeat sequences (‘TTAGGG’ in humans) at the end of chromosomes [1]. Telomerase has the ability to overcome replicative senescence and has been shown to be active in 80–90% of cancers [2]. Telomerase has been hypothesized to function independently of DNA synthesis in somatic cells, although most somatic cells show little or no telomerase activity [3]. Telomerase regulates gene expression independently of its activity in the maintenance of telomeres through transcription factors such as BRG1, MYC, and NF-kB [4–6]. Telomerase recognizes the sequence ‘TTGGG,’ a predicted telomerase-responsive element, in promoter regions in a TERC-dependent manner and thus supports the transcriptional activation of target genes [6]. Recent reports show a relationship between telomerase and metabolism [4, 7, 8]. For instance, TERT stabilizes MYC and thus activates or inhibits targets of MYC such as GAPDH, hexokinase 2 (HK2), and PKM2 [4]. Furthermore, studies show that the deletion of TERT or TERC inhibits autophagy activation and alters mammalian target of rapamycin (mTOR) activity in various cell lines and in mouse kidneys [9, 10]. Although independent investigations show that telomerase activates autophagy, the underlying molecular mechanisms are unclear.

Macroautophagy (herein autophagy) is an evolutionarily conserved catabolic process that degrades organelles and cytosolic components in lysosomes [11]. Autophagy occurs at low levels under normal conditions and is further activated by various stresses, such as starvation [12]. In cancer cells, autophagy is required for survival in nutrient-restricted and stressful conditions [13]. One of the most well characterized central regulators of autophagy is mTOR [14, 15]. Under normal conditions, mTOR regulates cellular physiology by phosphorylating S6K, 4E-BP, and ULK1 [16]. The activity of mTOR is affected by mediators of nutrient-sensing and metabolic pathways, such as RAPTOR, AKT, AMPK, and HK2 [17, 18]. mTOR activity is inhibited by rapamycin treatment, amino acid deprivation, and glucose starvation, all of which activate autophagy [19–21].

HK2 is an essential glycolysis enzyme that catalyzes the first committed step in glycolysis from glucose to glucose-6-phosphate. Although HK2 is highly expressed in embryonic tissues, it is mainly expressed in adipocytes and myocytes in adults [22]. Cancer cells frequently show abnormal expression of HK2 [23–25], which leads to the production of lactate even under aerobic conditions, known as the Warburg effect [26]. Under glucose-starvation conditions, HK2 promotes the transition from glycolysis to autophagy by inhibiting mTOR [18]. Similarly, treatment with 2-deoxyglucose (2-DG), an HK inhibitor, inhibits autophagic flux through the HK2-mTOR axis [18]. Although the mechanism by which HK2 activates autophagy has been studied [18], its upstream regulators remain unexplored.

Here, we show that telomerase regulates autophagy through HK2. We found that telomerase-induced HK2 activation attenuates mTOR activity, thereby activating autophagy. Furthermore, we found that telomerase binds to the HK2 promoter at a specific ‘TTGGG’ sequence through the telomere-responsive element of TERC. Thus, we establish a nexus among those signaling molecules, forming a telomerase-HK2-mTOR-autophagy axis.

## Materials and methods

### Vector constructs and mutagenesis

Human TERT was previously described by our group [27]. The HK2 construct was purchased from Addgene. Dr. J.M. Lee (College of Medicine, Yonsei University) provided the HK2 promoter-Luc construct. Dr. L. Joachim (Ecole Polytechnique Fédérale de Lausanne) provided the human TERC-expressing construct. The mutant TERC construct was generated by PCR-based mutagenesis [28] with the following primers: 5’-GTC TAA GGC TTA CTG AGA AGG GCG-3’ and 5’-TCT CAG TAA GCC TTA GAC AAA AAA TG-3’. shRNA-resistant TERT was generated by PCR with the following primers: 5’- CTG TTA CAG CAT ACT GAA AGC CAA GAA CGC AGG GA-3’ and 5’- GTA TGC TGT AAC AGA GGG AGG CCG TGT CAG AGA TG-3’. Double-stranded control siRNAs and siRNAs against HK2, TERC, and TERT were purchased from Cosmo Genetech, Inc. The sequences of all constructs were confirmed (Cosmo Genetech, Inc.) prior to use.

### Cell culture, transfection, and establishment of stable cell lines

HepG2 (ATCC), A549 (ATCC), MCF-7 (ATCC), and HeLa (Korean Cell Line Bank) cells were cultured and maintained in RPMI 1640 (Gibco) or Dulbecco’s Modified Eagle’s Medium (DMEM; Gibco) supplemented with 10% fetal bovine serum (FBS; Sigma) and 1% penicillin/streptomycin (Gibco) at 37°C in a humidified chamber containing 5% CO_2_. Cells were grown in DMEM or glucose-deprived DMEM (Gibco) supplemented with 10% dialyzed FBS (Sigma) and 1% penicillin/streptomycin for the indicated amounts of time with or without bafilomycin (Sigma) or rapamycin (Sigma). To establish stable cell lines, lentiviruses encoding shRNA (Sigma) were transduced into HepG2 cells in the presence of polybrene (Sigma), and cells were selected with puromycin (1 μg/mL, Sigma). The lentiviruses were produced and manipulated as previously described [29]. Plasmid and siRNA transfection were carried out using Lipofectamine 3000 and Lipofectamine RNAiMAX (Invitrogen), respectively, as recommended by the manufacturer.

### Immunocytochemistry and LC3 puncta assays

Cells were transfected with RFP-GFP-LC3 and seeded on gelatin-coated coverslips. Following fixation in 4% paraformaldehyde in phosphate-buffered saline, the cells were permeabilized with 0.5% Triton X-100 (Sigma) to allow penetration of DAPI (Sigma). The cells were analyzed using a Laser Scanning Microscope (LSM880, Carl Zeiss). LC3 dots were quantified using ImageJ software. All analyses were performed as previously described [9, 30].

### ChIP assays

ChIP assays were performed as previously described [29]. PCR was performed with the following primers: 5’-AGA GAA GGA GTA AGA CAA GG-3’ (-2 kb TTGGG-Fwd), 5’-CAT CTA GCC TCT CAG CAA-3’ (-2 kb TTGGG-Rvs), 5’-CAG GGA CTA CAG GCA CAT-3’ (2^nd^ TTGGG-Fwd), 5’-ATG AGG AAA TTG AGG CAA GG-3’ (2^nd^ TTGGG-Rvs), 5’-GTG TCA CCC AGC TAA GAA A-3’ (TGGG-Fwd), and 5’-GCG TCA CAA CTG CTA GAA-3’ (TGGG-Rvs).

### Media glucose and lactate levels

Glucose and lactate levels in the culture medium were measured using colorimetric assay kits (Sigma for glucose; Abcam for lactate) according to the manufacturers’ instructions.

### Western blotting and RT-qPCR

Protein lysates were analyzed by Western blot with antibodies specific for TERT (Novus), HK2 (Santa Cruz), GAPDH (Santa Cruz), ACTIN (Santa Cruz), HA (Santa Cruz), LC3 (Sigma), S6K (Cell Signaling), or p-S6K (Cell Signaling). Total RNA was isolated with Trizol (Invitrogen) according to the manufacturer’s instructions. Superscript III (Invitrogen) was used for cDNA synthesis as previously described [31]. iQ5 (Bio-Rad) was used for RT-qPCR with the following primers: human *HK2*, 5’-GAG TTT GAC CTG GAT GTG GTT GC-3’ and 5’-CCT CCA TGT AGC AGG CAT TGC T-3’; human *TERT*, 5’-GCC GAT TGT GAA CAT GGA CTA CG-3’ and 5’-GCT CGT AGT TGA GCA CGC TGA A-3’; human *TERC*, 5’-GGC CAT TTT TTG TCT AAC CC-3' and 5’-GTT TGC TCT AGA ATG AAC GG-3'; and human *ACTIN*, 5’-CAC CAT TGG CAA TGA GCG GTT C-3’ and 5’-AGG TCT TTG CGG ATG TCC ACG T-3’.

### Luciferase assays

Luciferase assays were performed as previously described [28]. Mutant HK2 promoter-Luc constructs of the ‘TTGGG’ sequence were generated by PCR-mediated point mutagenesis as previously described [28] with the following primers: HK2 TAGCC, 5’-GCT ATT AGC CAC GCT GAA GTG GGA CTG-3’ and 5’-AGC GTG GCT AAT AGC TGG GAT TAC AAG GGC-3’; HK2 promoter TTAGGG mutant, 5’-GCT ATT TAG GGA CGC TGA AGT GGG AGG ACT G-3’ and 5’-AGC GTC CCT AAA TAG CTG GGA TTA CAA GGG CG-3’. HK2 promoter constructs for the deletion mutant were generated by PCR with the following primers: 5’-AAA AAG CTT TGC CAG TTT CCA CTG TGT GT-3’ (-1.7 kb-Fwd), 5’-AAA AAG CTT GAT CCC GAG ATG CCA GAC AG-3’ (-0.8 kb-Fwd), and 5’-AAA CTC GAG AAG CAG ATG CGA GGC AAT CA-3’ (HK2 promoter-Rvs). The PCR constructs were subcloned into pGL3-basic (Promega). pRL-*tk*-luc (Promega) was co-transfected with an HK2 promoter-luciferase construct used as a control.

### Statistical analysis

Statistical significance was determined using two-tailed Student’s t-tests. Data were analyzed using GraphPad Prism (GraphPad Software). *P*-values less than 0.05 were considered statistically significant.

## Results

### TERT activates autophagy

**Fig 1:**
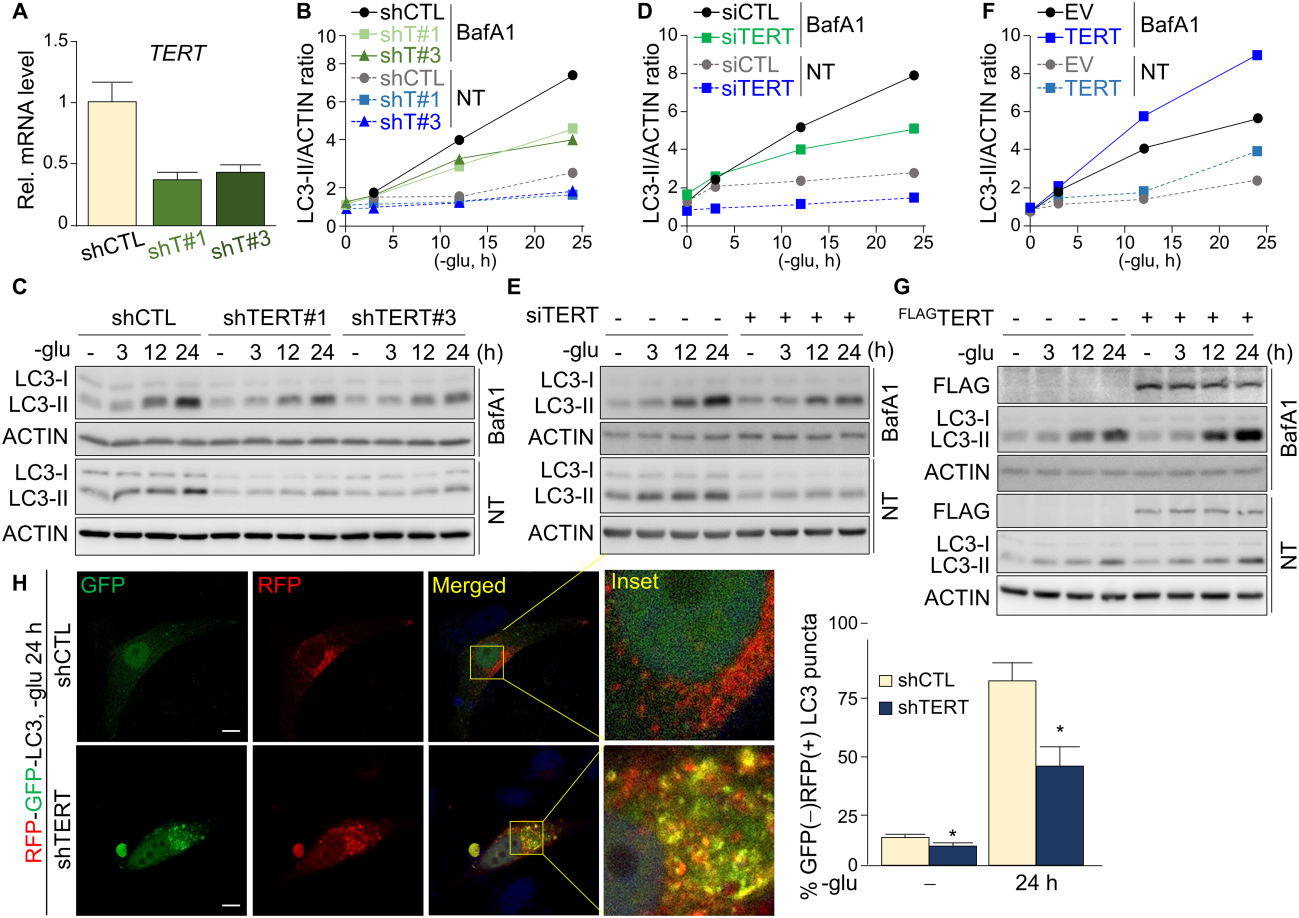
TERT activates autophagy under normal and glucose-starvation conditions. (**A**) Relative *TERT* mRNA levels in control (shCTL) and TERT knockdown (KD) HepG2 cells (shT#1 and shT#3; triplicate). (**B–G**) Quantification and Western blot analysis of the LC3 maturation rate in control and TERT KD HepG2 cells under normal and glucose deprivation conditions. ACTIN was used as a loading control in the Western blots. Cells were starved of glucose and treated simultaneously with bafilomycin A1 (BafA1, 10 μM) or vehicle (NT). (**B** and **C**) The LC3 maturation rate in control and stable TERT KD HepG2 cells under glucose deprivation. (**D** and **E**) The LC3 maturation rate in control and siRNA-mediated transient TERT KD (siTERT) HepG2 cells under glucose deprivation. (**F** and **G**) The LC3 maturation rate in empty vector control (EV) and TERT-overexpressing (TERT) HepG2 cells under glucose deprivation. (**H**) Representative images of immunocytochemistry analysis of LC3 puncta in control and TERT KD (shTERT) HepG2 cells transfected with RFP-GFP-LC3 (left panel). Scale bar: 10 μm. The percentage of GFP(-)RFP(+) LC3 puncta were measured and normalized with the number of GFP(+)RFP(+) LC3 puncta under normal and 24 h glucose-starvation conditions (right panel). The graph represents the normalized GFP(-)RFP(+) puncta per cell. (**B-G**) Bafilomycin A1 was treated to promote accumulation of LC3-II by blocking its degradation. Error bars indicate standard deviation. *p < 0.05.

Telomerase deficiency impairs glucose metabolism and autophagy [7, 10]. In addition, caloric restriction increases telomerase activity and enhances autophagy [32], suggesting links among glucose metabolism, telomerase, and autophagy. However, the specific contribution of telomerase to the autophagy process remains largely unknown. To assess the functional interaction between telomerase and autophagy, we deprived control and TERT knockdown (KD) HepG2 cells (Fig 1A) of glucose for 3, 12, or 24 h. To clearly check the autophagy activation throughout the time points, we examined the LC3-II levels under bafilomycin A1 treated conditions, which inhibits the last step of autophagolysosome formation, resulting in suppression of LC3 turnover [33]. Glucose deprivation-induced LC3 maturation (LC3-II) was impaired in stable (Fig 1B and 1C) and transient (Fig 1D and 1E) TERT KD cell lines compared with that in control HepG2 cells. Overexpression of shRNA-resistant TERT rescued the impaired autophagy activation in the TERT KD HepG2 cell line (Panel A in S1 Fig). Consistently, TERT overexpression accelerated the accumulation of LC3-II in HepG2 cells (Fig 1F and 1G). To further confirm those results, we overexpressed a red fluorescent protein (RFP) and green fluorescent protein (GFP) double-tagged LC3 construct in HepG2 cells to check mature and immature LC3 puncta [33–35]. Because the GFP signal is sensitive to acidic conditions, whereas the RFP signal is resistant to such conditions, immature autophagosomes show yellow dots due to intact GFP and RFP signals, but mature autophagolysosomes show only the RFP signal. After 24 h or glucose starvation, mature autophagolysosomes (GFP-negative and RFP-positive signal, GFP(-)RFP(+)) were mainly formed in the control cells, whereas immature autophagosomes (GFP and RFP double positive signal, GFP(+)RFP(+)) were evident in the TERT KD HepG2 cells (Fig 1H). In addition, we similarly detected TERT-mediated autophagy activation in three additional cell lines (MCF-7, A549, and HeLa; Panels B–D in S1 Fig). Those results indicate that TERT increased autophagy activity in the normal condition and further enhances under glucose-starvation condition in HepG2, MCF-7, A549, and HeLa cells.

### TERT promotes glucose consumption, lactate production, and HK2 expression

**Fig 2:**
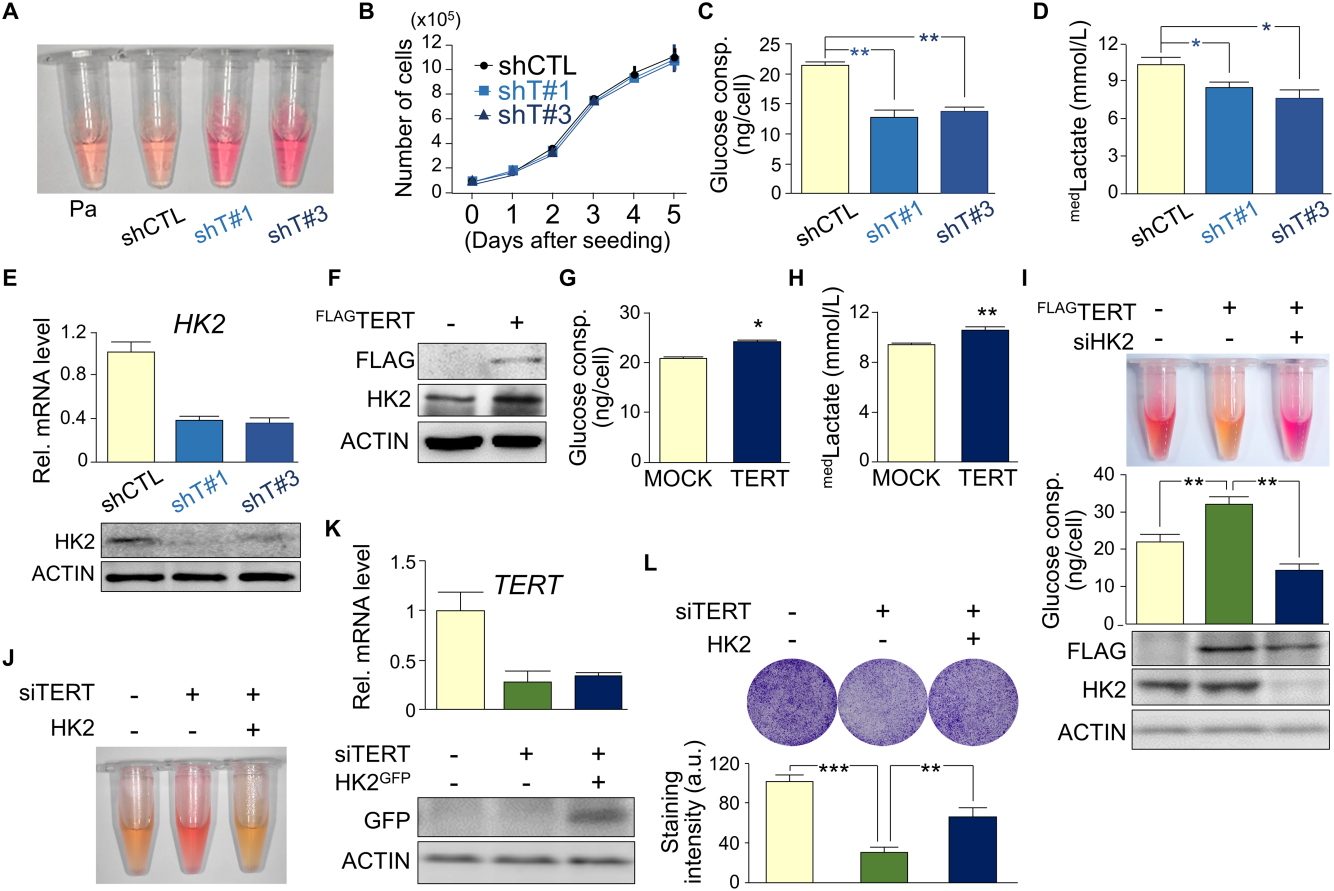
TERT activates HK2 expression and glucose consumption. (**A**) Representative images of the color of the growth media 48 h after seeding with 3 × 10^5^ PA, shCTL, shT#1, or shT#3 cells. (**B**) Growth curves of control and TERT KD cell lines (*n* = 3). (**C–E**) Glucose consumption per cell (**C**), media lactate levels (**D**), and relative HK2 transcript and protein levels **(E**) of control (shCTL) and TERT KD (shT#1 and shT#3) cells (*n* = 3). (**F**–**H**) Western blot analyses for FLAG (TERT), HK2, and ACTIN (**F**), glucose consumption per cell (**G**), and media lactate levels (**H**) 48 h after transient transfection (*n* = 3). (**I**) Representative images of the color of the growth media (upper), quantification of glucose consumption (middle; *n* = 3), and FLAG, HK2, and ACTIN protein expression (lower) 48 h after transfection. (**J** and **K**) Representative image of media color 48 h after cell seeding (3 × 10^5^ cells) (**J**) and relative *TERT* and HK2-GFP mRNA (*n* = 3) and protein levels (**K**). ACTIN was used as a loading control for the Western blot. (**L**) Representative image (upper) and quantification of cresyl violet-stained cells (lower, *n* = 3) 72 h after siRNA transfection. Error bars indicate standard deviation. *p < 0.05; **p < 0.01.

To examine the molecular mechanism by which TERT regulates autophagy, we first examined physiological changes in control and TERT KD HepG2 cells. The TERT KD cells produced less acidification of the culture media compared with control cells (Fig 2A), while there were no differences in growth between the cell lines (Fig 2B). Analyses of glucose and lactate levels in the media, which affect acidification [36], revealed lower rates of glucose consumption and lactate production by the TERT KD cells compared with those by the control cells (Fig 2C and 2D). As HK2 promotes lactate production from glucose during glycolysis and is an indirect downstream target of TERT [4, 37], HK2 could be a target for TERT-induced metabolic changes in HepG2 cells. Real-time quantitative reverse-transcription polymerase chain reaction (RT-qPCR) and Western blot analyses showed a dramatic reduction of HK2 expression in the TERT KD cells (Fig 2E). Conversely, transient overexpression of TERT increased HK2 expression, glucose consumption, and lactate production (Fig 2F–2H) and indicated that acidification of the growth medium was blocked by the HK2 KD (Fig 2I). Additionally, transient KD of TERT by siRNA suppressed media acidification and cell growth, and those effects were partially blocked by HK2 overexpression (Fig 2J–2L), indicating that TERT regulates glucose metabolism through HK2. The contrasting findings that cell growth was attenuated by transient TERT KD but not by stable TERT KD could be explained by a lower TERT KD efficiency in the stable KD cells or by probable compensatory cell-growth signaling that is induced by the long-term depletion of TERT but not by the transient depletion of TERT.

### The HK2-mTOR pathway is required for TERT-mediated autophagy regulation

**Fig 3:**
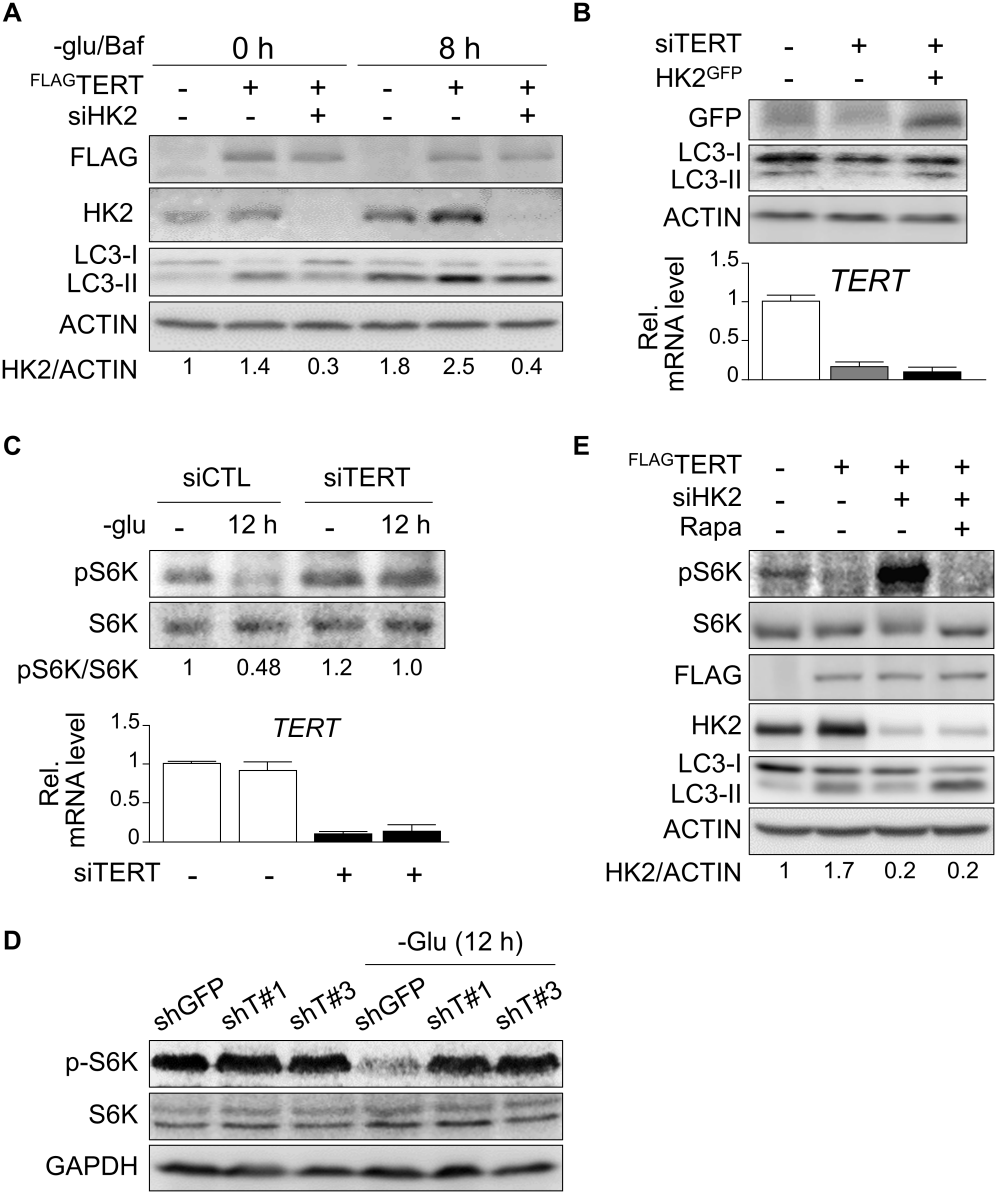
HK2 and mTOR are required for TERT-induced autophagy activation. (**A**) Western blot analyses for FLAG, HK2, LC3, and ACTIN 72 h after siRNA transfection. The times 0 h and 8 h indicate how long the cells were deprived of glucose and simultaneously treated with bafilomycin A1 (10 μM). (**B**) Western blot analyses for GFP, LC3, and ACTIN 72 h after siRNA transfection. (**C**) Western blot analyses for phospho-S6K (pS6K) and S6K72 h after siRNA transfection (**C**, upper panel) and knockdown efficiency of TERT (**C**, lower panel) in control (siCTL) and TERT KD (siTERT) cells after 0 h or 12 h of glucose starvation. (**D**) Western blot analyses for phospho-S6K (pS6K), S6K, and GAPDH in control and stable TERT KD HepG2 cell lines after 0 h or 12 h of glucose starvation. (**E**) Western blot analyses of pS6K, S6K, FLAG, HK2, LC3, and ACTIN 48 h after transfection in cells treated with either rapamycin (25 nM) or DMSO for 3 hours.

HK2 causes aerobic glycolysis and enhances autophagy by directly inhibiting mTOR activity [18, 38]. As TERT deficiency impairs autophagy and alters mTOR activity [10], we speculated that a TERT-mediated increase in HK2 expression could be responsible for autophagy activation. Indeed, TERT-induced acceleration of autophagy by glucose restriction was impaired by siRNA-mediated HK2 KD (Fig 3A). Conversely, the TERT KD-mediated inhibition of autophagy was rescued by HK2 overexpression (Fig 3B), suggesting that HK2 is required for TERT-induced autophagy activation. To examine the involvement of mTOR in the TERT-HK2-autophagy axis, we assessed mTOR activity by examining phosphorylation of ribosomal protein S6 kinase (S6K). Similar to a previous report [10], both transient and stable TERT KD impaired the glucose starvation-induced inhibition of mTOR activity (Fig 3C and 3D). Conversely, the TERT-mediated inhibition of mTOR activity was impaired by HK2 KD, and that effect was blocked by treatment with the mTOR inhibitor rapamycin (Fig 3E), suggesting that the mTOR pathway is regulated by TERT in an HK2-dependent manner. Furthermore, as expected, lower mTOR activity was associated with greater autophagic flux (Fig 3E). Thus, our results suggest that TERT activates glucose starvation-induced autophagy through the HK2-mTOR pathway.

### TERC activates autophagy through the HK2-mTOR pathway

As TERT forms a complex with TERC, and both TERT and TERC have been reported to regulate autophagy in the kidney [10], we surmised that TERC also contributes to autophagy activation during glucose starvation. Similar to TERT, TERC overexpression activated autophagy, as evidenced by LC3-II levels, in both normal and glucose-starved conditions, while siRNA-mediated KD of TERC suppressed autophagy (Panels A–E in S2 Fig). TERC overexpression also increased HK2 mRNA and protein expression and facilitated glucose consumption and lactate production (Panels F–I in S2 Fig). Furthermore, the TERC-induced activation of autophagy was suppressed by siRNA-mediated HK2 KD (Panel J in S2 Fig). TERC overexpression inhibited mTOR activity, as shown by reduced S6K phosphorylation and increased LC3-II levels, and those effects were blocked by HK2 KD (Panel K in S2 Fig). Moreover, rapamycin treatment reversed the HK2 KD-induced S6K phosphorylation and autophagy (Panel K in S2 Fig), indicating that TERC activates autophagy through the HK2-mTOR axis.

### TERT binds to the ‘TTGGG’ sequence in the HK2 promoter through TERC

**Fig 4:**
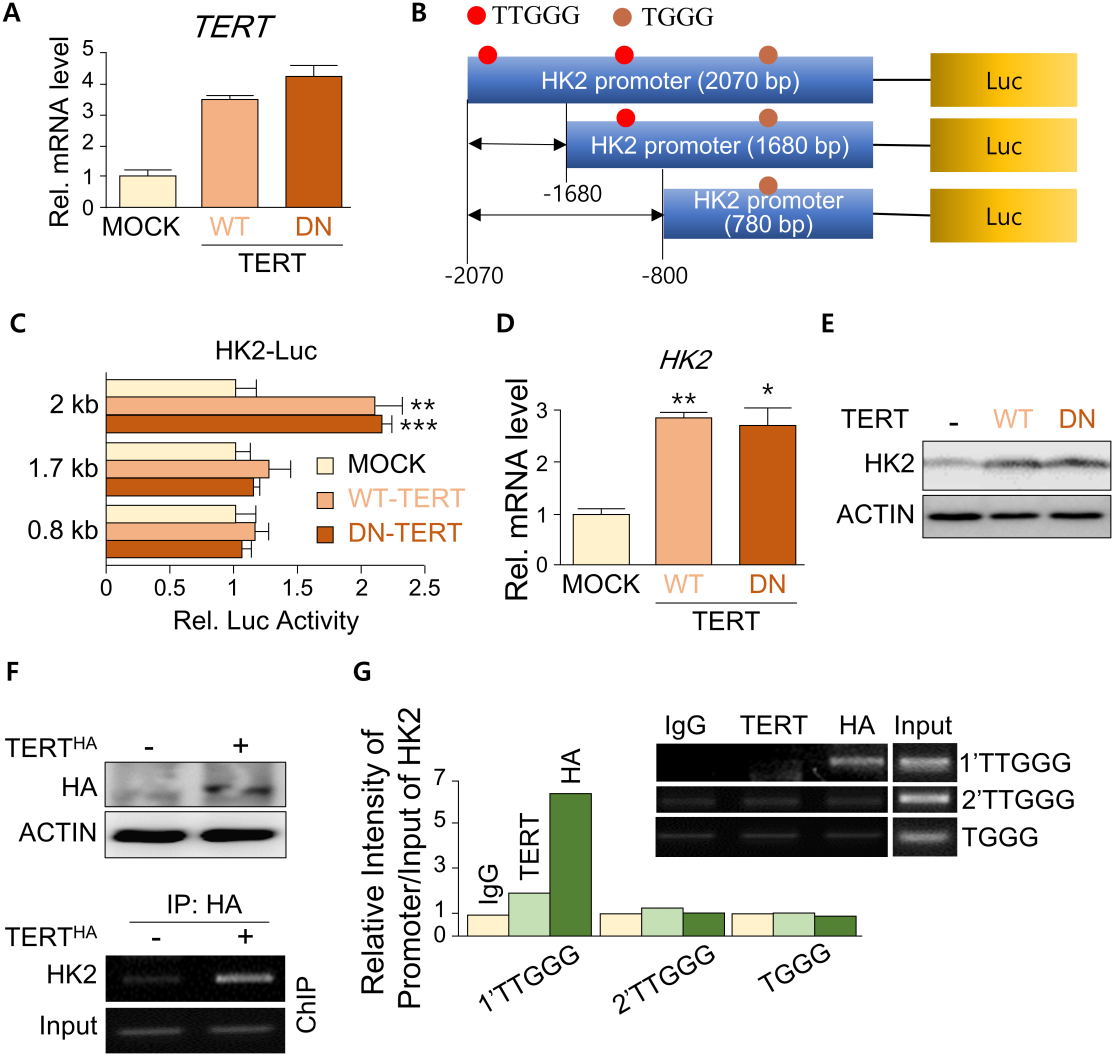
TERT binds to a TTGGG sequence on the HK2 promoter. (**A**) Relative *TERT* mRNA levels 48 h after transfection (n = 3) in HepG2 cells. (**B**) Schematic views of luciferase constructs harboring HK2 promoter with indicated length. Red spots indicate the sites of the ‘TTGGG’ sequences in the HK2 promoter. Brown spots indicate the sites of ‘TGGG’ sequences in the HK2 promoter. (**C**) Relative luciferase activity of 2, 1.7, and 0.8 kb-length HK2 promoters 24 h after ectopic overexpression of control vector (MOCK), wild-type (WT-TERT), and DN-TERT (triplicate) in HepG2 cells. HK2-luciferase activity was normalized based on pRL-tk-Luc. (**D** and **E**) RT-qPCR in triplicate (**D**) and Western blot (**E**) analyses for HK2 and ACTIN 48 h after transfection in HepG2 cells. (**F** and **G**) Western blot analysis for TERT and ACTIN (**F**, upper panel), and ChIP analyses of TERT-expressing cells with the indicated antibodies against the HK2 promoter (**F**, lower panel; **G**) 48 h after transfection in HepG2 cells. Error bars indicate standard deviation. **p < 0.01; ***p < 0.001.

As we found that both TERT and TERC regulate HK2 expression, we presumed that telomerase regulates HK2 expression at the transcriptional level. Luciferase and immunoblot analyses of the HK2 promoter showed that the overexpression of TERT or TERC enhanced HK2 promoter activity and mRNA expression (Fig 4A–4E, Panels A–C in S3 Fig) and that the simultaneous expression of both TERT and TERC had a synergistic effect on HK2 expression (Panels D and E in S3 Fig). As one of the major roles of TERT is telomere maintenance, we tested whether telomerase activity is involved in the regulation of HK2 expression. Similarly to that of wild-type (WT) TERT, the ectopic overexpression of a telomerase activity-defective form of TERT (DN-TERT) [39] increased HK2 promoter activity and gene expression (Fig 4C–4E). Therefore, in agreement with a previous report [4], our results suggest that telomere elongation is not important for TERT-induced HK2 expression. However, as TERC can recognize the specific sequence ‘TTGGG’ in promoter regions [6], we suspected that TERC could play a telomerase activity-independent role in HK2 transcriptional activation. Chromatin immunoprecipitation (ChIP) analyses showed that TERT could bind to a specific ‘TTGGG’ sequence, but not to other ‘TTGGG’ or ‘TGGG’ sequences, in the HK2 promoter (Fig 4F and 4G, S4 Fig), suggesting that TERT recognizes the HK2 promoter at the specific region and activates HK2 expression at the transcriptional level. Thus, TERC may participate in TERT-mediated HK2 transcriptional activation. To confirm that recognition of a ‘TTGGG’ sequence by TERC is required for HK2 expression, we conducted site-directed mutagenesis of the telomere binding motif (5’-CCCUAA-3’ to 5’-GGCUUA-3’) of TERC and its recognition motif (5’-TTGGG-3’ to 5’-TAGCC-3’) in the HK2 promoter. In contrast to WT TERC, the mutant TERC did not induce HK2 promoter activity or mRNA expression (Panels A–C in S3 Fig). In addition, the mutation of the recognition motif in the HK2 promoter reduced the responsiveness of the promoter to TERT (Panels F–H in S3 Fig). Furthermore, the alteration of the ‘TTGGG’ motif to the telomeric sequence ‘TTAGGG’ markedly increased the basal HK2 promoter activity, although similar levels of promoter activity between sequences were observed under TERT overexpression (Panel I in S3 Fig). The HK2 promoter contains the ‘TTGGG’ sequence rather than the telomerase-response element ‘TTAGGG.’ The alteration of ‘TTGGG’ to ‘TTAGGG’ in the HK2 promoter increased the basal promoter activity, although the alteration did not affect the promoter activity in the TERT-overexpression condition, suggesting that the ‘TTGGG’ sequence reduces the sensitivity of the promoter for TERT binding. Consistent with those observations, siRNA-mediated KD of TERC prevented TERT-induced autophagy and HK2 transcriptional activation (Panel J in S3 Fig). Therefore, our results suggest that TERT regulates HK2 expression through the binding of TERC to a ‘TTGGG’ sequence in the HK2 promoter.

## Discussion

By connecting independent results based on glucose metabolism, telomerase activity, and autophagy, we found that telomerase enhances autophagy activity upon glucose starvation. Although TERT is known to decrease p-S6K levels in renal tissues [10] and cardiac cells [40], the specific mechanism by which TERT regulates downstream targets of mTOR is poorly understood. We propose that TERT regulates the mTOR signaling pathway by inducing HK2 expression. Furthermore, we showed that TERC is essential for TERT-mediated HK2 transactivation in a telomere-independent manner, indicating that telomerase serves extra-telomeric functions in the nucleus. Alternative modes of TERT-mediated mTOR regulation may exist, however, depending on cell types and stress conditions, as TERT can bind to mTOR and Raptor [41].

Injury- and amino acid restriction-induced autophagy can be suppressed by depletion of telomerase and we show that telomerase activates autophagy in the glucose-starvation condition [9, 42]. Alternatively, Beclin 1-mediated autophagy reduces telomerase activity suggesting that telomerase and autophagy are related vice versa [43]. The evidences imply that telomerase protects the cells from stresses by activating autophagy and telomerase itself can be suppressed by autophagy in a mean of negative feedback.

The maximum promoter activity in the TERT-overexpression condition was equivalent between promoters containing the ‘TTGGG’ and ‘TTAGGG’ sequences. Compared with normal conditions, cellular stress can upregulate TERT expression [44], which in turn upregulates HK2 expression. However, if the HK2 promoter harbors the ‘TTAGGG’ sequence rather than the ‘TTGGG’ sequence, TERT-mediated HK2 induction could have only a minor effect on autophagy, as the basal HK2 expression level is already high. By all means, TERT should recruit the transcription factors and coactivators for transcriptional activation of the target gene expression as it itself is not a transcription factor.

A bundle of evidences is showing the important relevance between telomerase and cancer. Through the proliferation, normal cells undergo progressive telomere shortening and finally stop the cell division [45]. By overexpression of telomerase, it protects the end of chromosomes, thereby unlimited number of cell division comes out. However, this event does not fully explain the oncogenic phenomenon of telomerase overexpression. Recent studies showed that TERT regulates transcription of tumorigenesis-, inflammation-, and metabolism-related genes through MYC and NF-kB [4, 6, 46, 47]. Through those and unknown factors, TERT may orchestrate the tumorigenic and autophagic pathways, resulting in further progression of diseases.

We showed that telomerase regulates autophagy, which implies that this process could be targeted for cancer therapy. Cancer cells manipulate cellular metabolism by inducing the expression of glycolytic enzymes and upregulating aerobic glycolysis, a process known as the Warburg effect. Furthermore, as cancer cells scavenge nutrients by inducing autophagy, the inhibition of telomerase activity could restrict the autophagy-mediated nutrient supply to cancer cells. Thus, anti-telomerase drugs might be used to manipulate cancer cell metabolism independently of classical mechanisms based on telomere length.

## Supporting information

S1 Fig TERT promotes autophagy in various cell lines. (**A**) Western blot analysis of FLAG, LC3, and ACTIN 48 h after transfection of control and TERT KD HepG2 cells with the shTERT-resistant TERT construct. The LC3-II per ACTIN protein level is represented below. (**B**–**D**) Western blot analyses of FLAG, LC3, and ACTIN in MCF-7 (**B**), A549 (**C**), and HeLa (**D**) cells 48 h after transfection with TERT construct. The cells were deprived of glucose for indicated number of hours. The LC3-II per ACTIN protein level is represented below.
(PDF)

S2 Fig TERC activates autophagy through the HK2-mTOR pathway. (**A**) Relative level of TERC transcript expression (n = 3). ACTIN was used for normalisation. (**B**) Western blot analyses of LC3 and ACTIN 48 h after transfection under glucose deprivation. The cells were simultaneously deprived of glucose and treated with bafilomycin A1 (10 μM) for 3 h. (**C**) Relative level of TERC transcript expression (n = 3) 48 h after siRNA transfection. ACTIN was used for normalisation. (**D**) Western blot analysis of LC3 and ACTIN 48 h after siRNA transfection. The cells were deprived of glucose for the indicated number of hours. (**E**) Relative level of TERC transcript expression (n = 3) 48 h after siRNA transfection. ACTIN was used for normalisation. (**F** and **G**) RT-qPCR (n = 3; **F**) and Western blot (**G**) analyses of HK2 and ACTIN 48 h after transfection. (**H** and **I**) Media glucose consumption (**H**) and lactate levels (**I**) 48 h after transfection (n = 3). (**J**) Western blot analyses of LC3, HK2, and ACTIN 48 h after siRNA transfection. The cells were deprived of glucose for 8 h. (**K**) Western blot analysis of pS6K, S6K, HK2, LC3, and ACTIN 72 h after siRNA transfection. The cells were treated with rapamycin (Rapa, 25 nM) or DMSO for 3 h. HepG2 cells were used for the experiment of the S2 Fig. Error bars indicate standard deviation. *p < 0.05; **p < 0.01.
(PDF)

S3 Fig TERC and a ‘TTGGG’ sequence on the HK2 promoter are important for TERT-mediated transcriptional activation of HK2. (**A**) Relative level of TERC transcript expression (triplicate). ACTIN was used for normalisation. (**B**) Relative luciferase activity of the HK2 promoter 24 h after transfection (n = 3). (**C**) RT-qPCR (upper, n = 3) and Western blot (lower) analyses of HK2 and ACTIN 48 h after transfection. (**D**) Western blot analysis of HK2, TERT, LC3, and ACTIN 48 h after transfection. (**E**) Relative luciferase activity of the HK2 promoter 48 h after transfection (n = 3) (**F**-**H**) Relative luciferase activity of the wild type (WT) and ‘TAGCC’-mutant HK2 promoters 24 h after TERT (**F**) and 48 h after siTERT (**G**) transfection (n = 3). TERT expression levels were analysed by Western blot (**F**, lower panel) and RT-PCR (**H**). (**I**) Relative luciferase activity of the WT and ‘TTAGGG’-mutant HK2 promoters 24 h after transfection (n = 3). TERT expression was analysed by Western blot (**I**, lower panel). (**J**) Western blot analysis of LC3, HK2, FLAG, and ACTIN 72 h after siRNA transfection. HepG2 cells were used for the experiment of the S3 Fig. Error bars indicate standard deviation. *p < 0.05; **p < 0.01; ***p < 0.0001.
(PDF)

S4 Fig The promoter region of HK2 with TTGGG and its related sequences. The full length of human HK2 promoter used for the study. TTGGG and TGGG sequences are indicated in red color. The TTGGG sequence in the black box is responsible to telomerase. Translation start site is indicated in blue color.
(PDF)
