# Supplementary figures and images for: Hexokinase 2 is a molecular bridge linking telomerase and autophagy
Source: PLoS One. 2018 Feb 20;13(2):e0193182. doi: 10.1371/journal.pone.0193182 (PMC5819818; doi:10.1371/journal.pone.0193182)

# S1 Fig

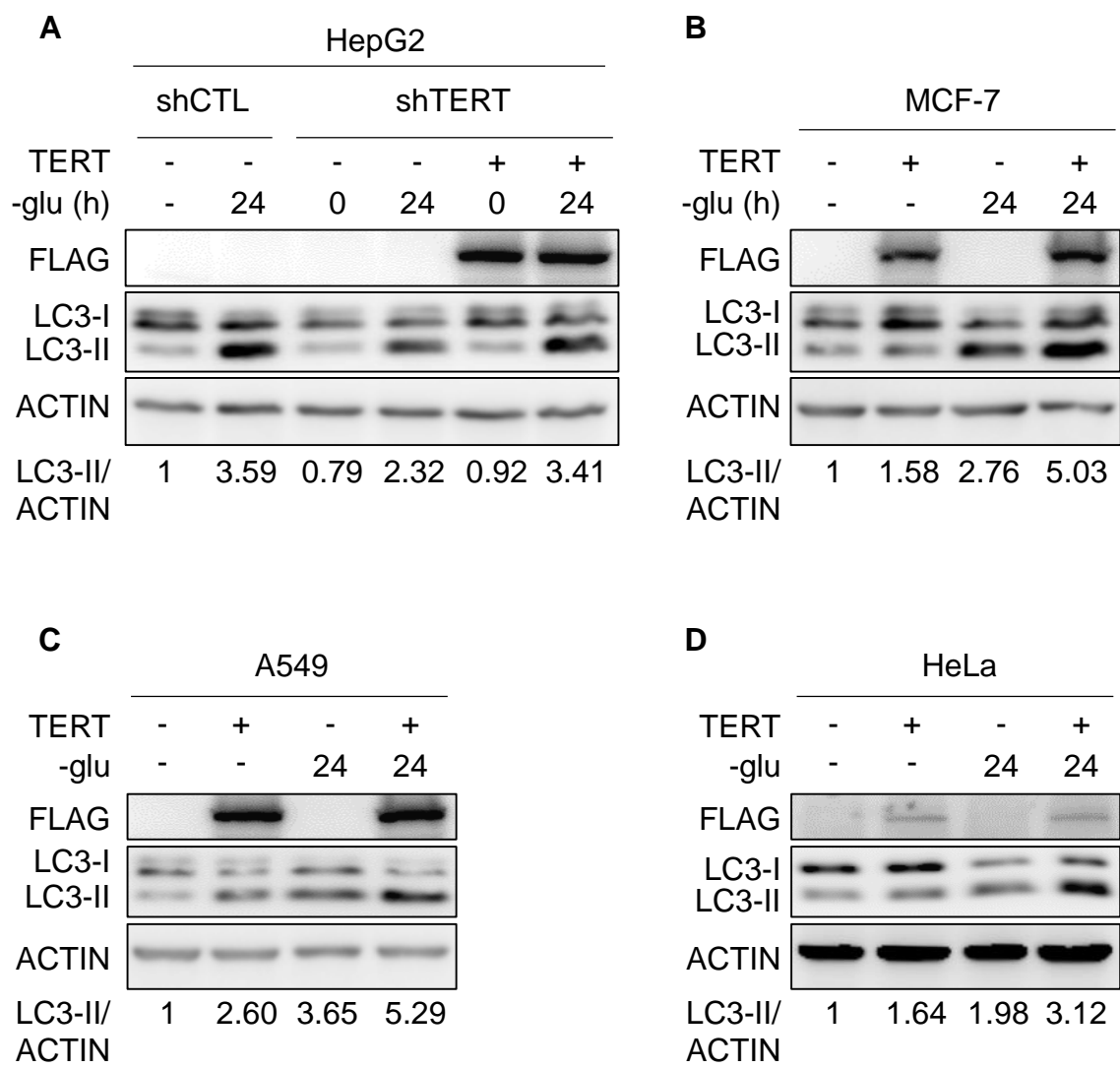

Supplement: S1 Fig — (A) Western blot analysis of FLAG, LC3, and ACTIN 48 h after transfection of control and TERT KD HepG2 cells with the shTERT-resistant TERT construct. The LC3-II per ACTIN protein level is represented below. (B–D) Western blot analyses of FLAG, LC3, and ACTIN in MCF-7 (B), A549 (C), and HeLa (D) cells 48 h after transfection with TERT construct. The cells were deprived of glucose for indicated number of hours. The LC3-II per ACTIN protein level is represented below. (PDF) [file pone.0193182.s001.pdf]

# S2 Fig

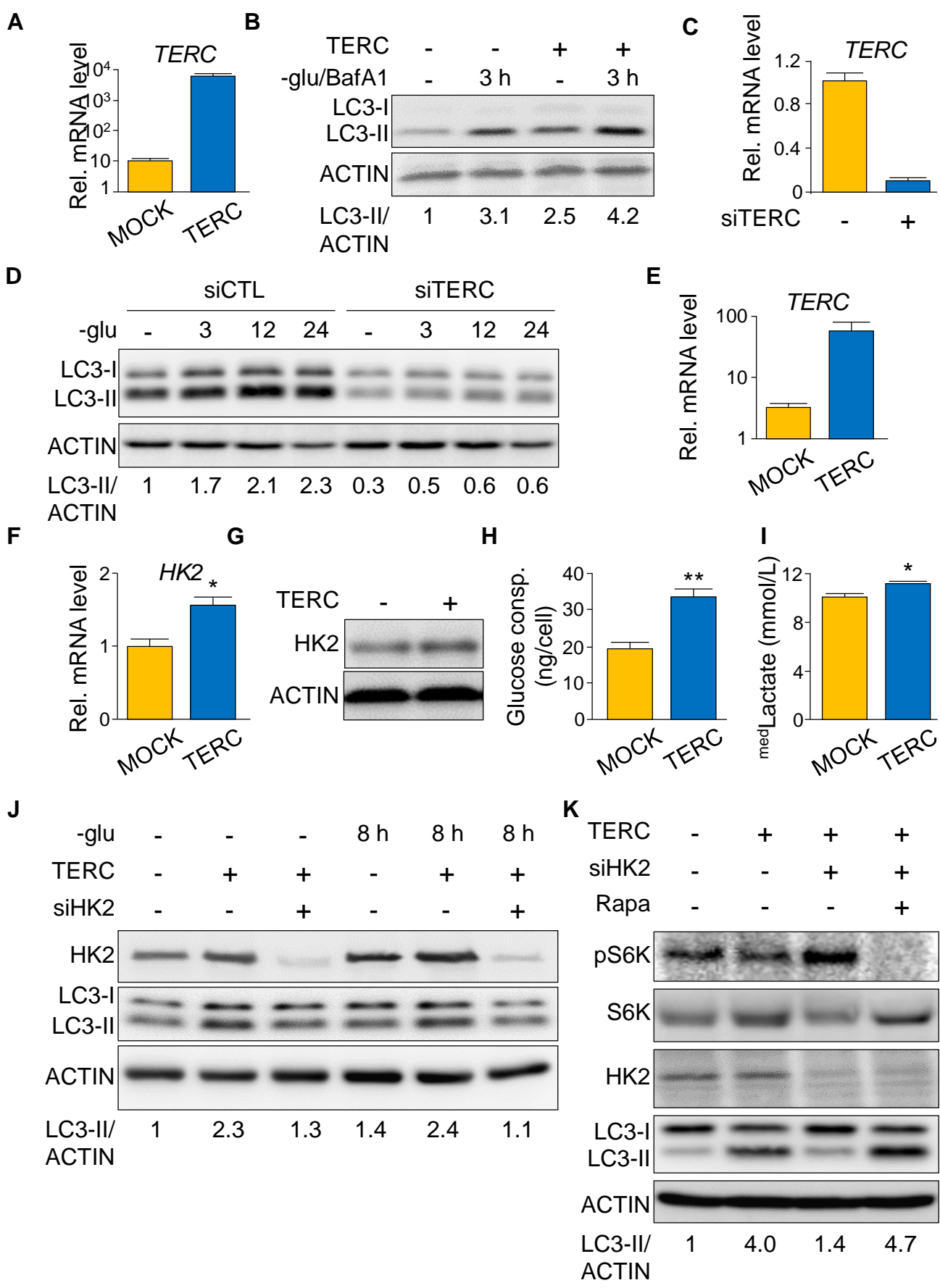

Supplement: S2 Fig — (A) Relative level of TERC transcript expression (n = 3). ACTIN was used for normalisation. (B) Western blot analyses of LC3 and ACTIN 48 h after transfection under glucose deprivation. The cells were simultaneously deprived of glucose and treated with bafilomycin A1 (10 μM) for 3 h. (C) Relative level of TERC transcript expression (n = 3) 48 h after siRNA transfection. ACTIN was used for normalisation. (D) Western blot analysis of LC3 and ACTIN 48 h after siRNA transfection. The cells were deprived of glucose for the indicated number of hours. (E) Relative level of TERC transcript expression (n = 3) 48 h after siRNA transfection. ACTIN was used for normalisation. (F and G) RT-qPCR (n = 3; F) and Western blot (G) analyses of HK2 and ACTIN 48 h after transfection. (H and I) Media glucose consumption (H) and lactate levels (I) 48 h after transfection (n = 3). (J) Western blot analyses of LC3, HK2, and ACTIN 48 h after siRNA transfection. The cells were deprived of glucose for 8 h. (K) Western blot analysis of pS6K, S6K, HK2, LC3, and ACTIN 72 h after siRNA transfection. The cells were treated with rapamycin (Rapa, 25 nM) or DMSO for 3 h. HepG2 cells were used for the experiment of the S2 Fig. Error bars indicate standard deviation. *p < 0.05; **p < 0.01. (PDF) [file pone.0193182.s002.pdf]

S3 Fig

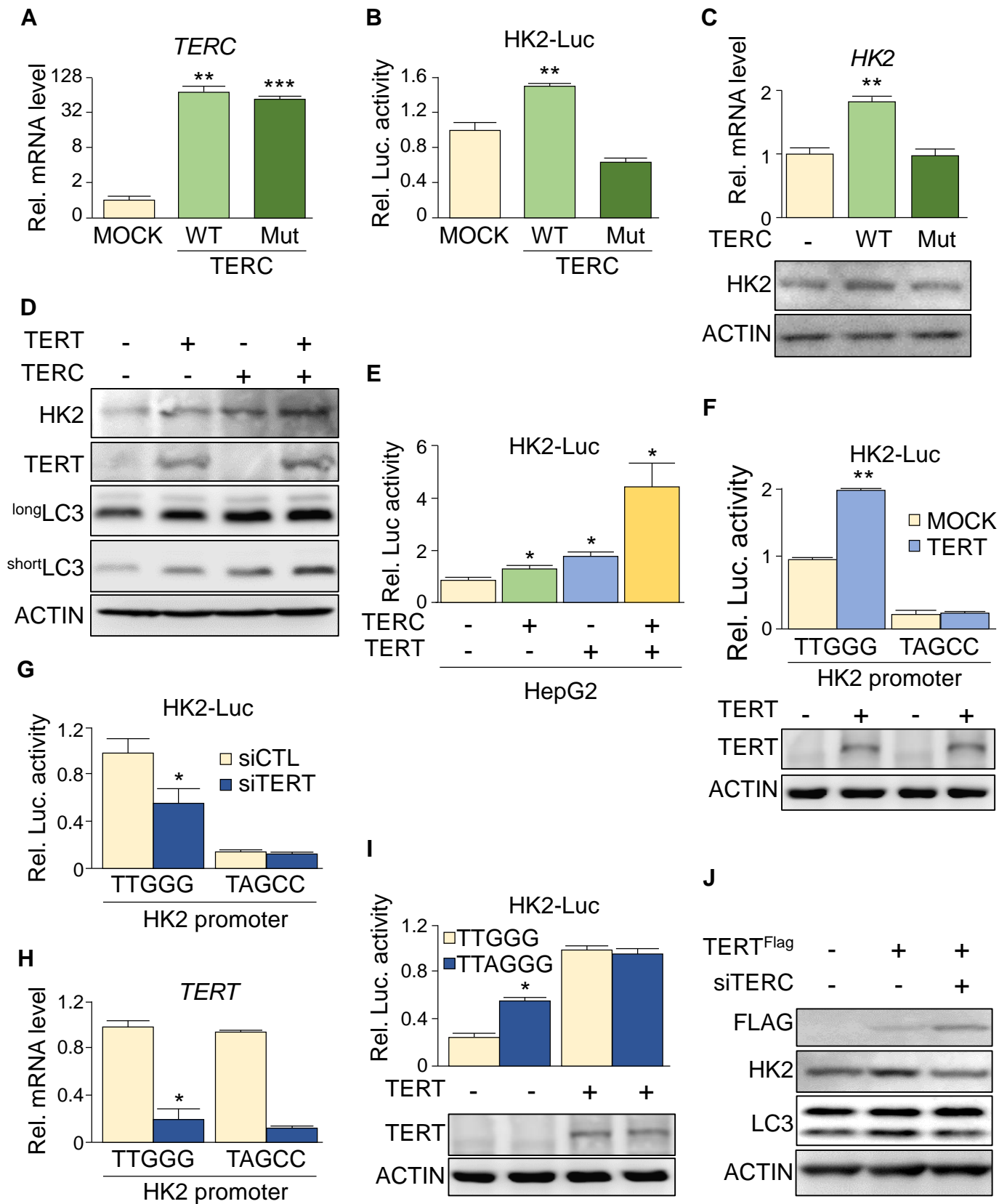

Supplement: S3 Fig — (A) Relative level of TERC transcript expression (triplicate). ACTIN was used for normalisation. (B) Relative luciferase activity of the HK2 promoter 24 h after transfection (n = 3). (C) RT-qPCR (upper, n = 3) and Western blot (lower) analyses of HK2 and ACTIN 48 h after transfection. (D) Western blot analysis of HK2, TERT, LC3, and ACTIN 48 h after transfection. (E) Relative luciferase activity of the HK2 promoter 48 h after transfection (n = 3) (F-H) Relative luciferase activity of the wild type (WT) and ‘TAGCC’-mutant HK2 promoters 24 h after TERT (F) and 48 h after siTERT (G) transfection (n = 3). TERT expression levels were analysed by Western blot (F, lower panel) and RT-PCR (H). (I) Relative luciferase activity of the WT and ‘TTAGGG’-mutant HK2 promoters 24 h after transfection (n = 3). TERT expression was analysed by Western blot (I, lower panel). (J) Western blot analysis of LC3, HK2, FLAG, and ACTIN 72 h after siRNA transfection. HepG2 cells were used for the experiment of the S3 Fig. Error bars indicate standard deviation. *p < 0.05; **p < 0.01; ***p < 0.0001. (PDF) [file pone.0193182.s003.pdf]
