# Supplementary material for: Hexokinase 2 is a molecular bridge linking telomerase and autophagy
Source: PLoS One. 2018 Feb 20;13(2):e0193182. doi: 10.1371/journal.pone.0193182 (PMC5819818; doi:10.1371/journal.pone.0193182)
Supplement: S4 Fig — The full length of human HK2 promoter used for the study. TTGGG and TGGG sequences are indicated in red color. The TTGGG sequence in the black box is responsible to telomerase. Translation start site is indicated in blue color. (PDF) [file pone.0193182.s004.pdf]

S4 Fig

TTGCTGAGCACATCTGTAATCCCAGCTAT**TTGGG**ACGCTGAAGTGGGAGGACTGCTTGAGCC
CAGGAGTTCAAGACCAGCCTGGGCAACATATCGAGTCCCTGCTTTAAAAAAAAAAAAAAGGA
AGTTTTGCTGAGAGGCTAGATGGATTATGATTTTTGTTTATTTTTCCTGTTTATCCATATAT
TATTTTTCAACAATGAGTATTGATTACTTATATAATAATTTTAAGGCTGTACACATTGCAGA
CAGCACCCCACTGTTTGAAAAACTCCTCCTCAGTAGAACATGGCAGACCTTCATCTTCCTTC
CCTGAACCTTTTCCAACCTTAGGCTTGCCATTCTCCACCAGTGCTAATGTCATGTCTCTTGA
AATCTGTATTGAAGTCAGTATTTCATTCTTGCCAGTTTCCACTGTGTGTTTAAATTTGGAGT
CTGGTGTCTAGCATTAGCTGGGGTTGGGGCTTCCACTCCTCTCAGCATTGGTAAGCCTCCTC
ACCCACCCCATCCCATGTCCAAGATCACCCAGTTACACACTTACCATCTACCCAGTTCATTC
ACATCATCAGTCCCAGAGCTGCAGAGATGCTCTTTTTCTACCTCCTACTTCTCTGGCTCTTA
GAGAGGCAGCATGGGATAATGGGGCAAGCGAATAGGGCCTTAAAGTAGAGGGACAAGGGTTC
TCTTCCCTATCTGCCACTTATTAGCTATGTGACCTCGTGTAAGTCTCTTTTCTTTTTGAGAC
AGGGTCTCCCTCTGTCACCTAGGCTGGAGTACAGTGGTATGATCATAGCTCACTGCAGCCTC
GAACTCCTGGGCTCAAGCTATCCTTCCACCTTAGCCTTCTGAGCAGCAGGGACTACAGGCAC
ATGCCACCATGTCCGGCTGATTTATTTATTTTTAT**TTGGG**AAGATGGGGGTCTCACTATGTC
GCCCAGGCTGGTCATGAACTCCTGGTCTCAAGCAACCCTCCAACCTTGGACTCCCAAAGTGC
TGGGATTACAGGTGTGAGCCCTGGCCTTGCCTCAATTTCCTCATCTGTAAAACGGGGTTAGT
GAAACTCACATCCTATCAGTGGTTTTGAGGATGGGCCGACTCTTGTATTGCCTGCTCTAGTA
CAATCAGCAGCTAAGGCGGCTCACTTTCCGGCCGTGCTACAATAGGTAAGAACTAGGATGCT
TTAGACGTGTGACTGGGCAGTGGGAGCCCCTCACATGATCCCGAGATGCCAGACAGTGTCTC
TCCGCACAGGGCGTGTGCTGGTCCAGAGGCCCGTTTTTCCAGTCGCCCCACACCCCGGGTCC
GCGATCACGCTCCCCCCACCCATAGCCGAGCCTGACGCGGCGGTGGCTCATGCGCCTTTCCG
TCCCAGCCTTTAGCCACGGACCACACGTCCCATCTCAGGCGCCCCGCCCCTCCCCCGCCCCC
CGCCCCCGGCGCGCCTCCCCAGGCTGCCGGCTCCGGTGTCTGAGCGGCCGCGCCCGCGAGCC
GTGAGCGATGATTGGCTGCGCCACGGCGGCGGGCGGTCCGTGGGCGCACACACCCTCCCCGC
GCAGCCAA**TGGG**CGTGCGCACGTCACTGATCCGGAGGCCCGCGGGCCGGCAGCCCCTCAATA
AGCCACATTGTTGCATGAAACTCCGGCGCAGGAGTCCCGGGCTGCCGCTGGCAACATCGTGT
CACCCAGCTAAGAAAATCCGCGGGCCCGAGCCACGCGCCTGTGAATCGGAGAGGTCCCACTG
CCCGAGTGGAGCCGGGCTGAGATTCTTCTCAAGTTGAGCCTCAGTGATCCTGTGGCCGAAGT
TAGCGCCTTGACGTGGGACAACCGGACACGTCGCCAGGAGAGAACTGAGGCGCCTTCTAGCA
GTTGTGACGCCAAAATCACGTCTCCGGAGACCCGCGCCCTCCGCCAGCCGGGCGCACCCTCG
CCGGTAGCCTTCTTTGTGCGCCGTCCGGACTCCCAGCTCCCGGCCCGGCAGCCGAGCCCCAG
CACAAAGCAGTCGGACCGCGCCGCCCGCCTCCCCTCTCGCGTCTCCGCCTCGGTTTCCCAAC
TCTGCGCCGTCGGGCCGCGGCAGG**ATGATTGCCTCGCATCTGCTTGCC**
